# Effects of Rotavirus Vaccination Coverage among Infants on Hospital Admission for Gastroenteritis across All Age Groups, Japan, 2011–2019

**DOI:** 10.3201/eid3009.240259

**Published:** 2024-09

**Authors:** Kenji Kishimoto, Susumu Kunisawa, Kiyohide Fushimi, Yuichi Imanaka

**Affiliations:** Graduate School of Medicine, Kyoto University, Kyoto, Japan (K. Kishimoto, S. Kunisawa, Y. Imanaka);; Tokyo Medical and Dental University Graduate School of Medicine, Tokyo, Japan (K. Fushimi);; Centre for Health Security, Graduate School of Medicine, Kyoto University, Kyoto (Y. Imanaka);

**Keywords:** gastroenteritis, rotavirus, enteric infections, viruses, bacteria, vaccination coverage, vaccines, hospitalization, Japan

## Abstract

We assessed the effect of rotavirus vaccination coverage on the number of inpatients with gastroenteritis of all ages in Japan. We identified patients admitted with all-cause gastroenteritis during 2011–2019 using data from the Diagnosis Procedure Combination system in Japan. We used generalized estimating equations with a Poisson distribution, using hospital codes as a cluster variable to estimate the impact of rotavirus vaccination coverage by prefecture on monthly numbers of inpatients with all-cause gastroenteritis. We analyzed 294,108 hospitalizations across 569 hospitals. Higher rotavirus vaccination coverage was associated with reduced gastroenteritis hospitalizations compared with the reference category of vaccination coverage <40% (e.g., for coverage >80%, adjusted incidence rate ratio was 0.87 [95% CI 0.83–0.90]). Our results show that achieving higher rotavirus vaccination coverage among infants could benefit the entire population by reducing overall hospitalizations for gastroenteritis for all age groups.

Gastroenteritis is one of the most common infectious diseases, characterized by rapid onset of diarrhea with or without nausea, vomiting, fever, and abdominal pain (*1*,*2*). Enteric viruses and bacteria are major causes of gastroenteritis (*3*,*4*), although causes of hospitalizations related to gastroenteritis are often unidentified (*5*). Gastroenteritis affects persons of all ages and is a leading cause of death worldwide (*6*,*7*); younger children, the elderly, and immunocompromised patients in particular are at risk of severe gastroenteritis (*8*–*11*). Gastroenteritis-related hospitalizations and outpatient visits continue to be significant burdens on health systems (*3*,*12*,*13*). 

Rotavirus is a leading cause of gastroenteritis and diarrhea, including fatal illnesses, in both young children and persons of all ages (*6*). A recent systematic review and meta-analysis reported that the pooled rotavirus prevalence was 7.6% (95% CI 6.2%–9.2%) among persons >5 years of age with diarrhea (*14*). Rotavirus infection is estimated to cause >200,000 deaths annually in children <5 years of age (*15*). Oral live-attenuated rotavirus vaccines for infants have been implemented worldwide since 2006. The World Health Organization in 2009 recommended routine immunization for all infants to prevent rotavirus disease (*16*). 

In Japan, the rotavirus vaccine was introduced in 2011 to be administered to infants 2–8 months of age. Rotavirus vaccination coverage in Japan gradually increased from 30% in 2012 to 78% in 2019; however, disparity among prefectures in coverage rates remains relatively high (≈30% in 2019) (*17*). Implementation of rotavirus vaccines has substantially reduced hospitalizations from rotavirus and all-cause acute gastroenteritis in infants and younger children both in Japan and in other countries (*18*–*20*). Of note, population-based surveillance for rotavirus gastroenteritis hospitalizations after introduction of rotavirus vaccination identified a reduction in age-specific hospitalization rates among children ineligible for rotavirus vaccination because they were above the upper age limit (*21*). Furthermore, a large study analyzing saved bacterial cultures from fecal samples reported an almost 50% decline in rotavirus prevalence among adults during the peak rotavirus season after the vaccine was introduced (*22*). Those studies suggest that rotavirus vaccination for infants might indirectly protect older children and adults from rotavirus.

The long-term impacts of rotavirus vaccination on the entire population have not been fully clarified. Given that gastroenteritis constitutes a significant health concern for person in all age groups, analyzing the effects of rotavirus vaccination on older age groups is warranted. Several recent studies investigating long-term trends after rotavirus vaccine introduction have shown the potential indirect protection of rotavirus vaccination against gastroenteritis (*23*,*24*). However, those findings of indirect protection in adults lack consistency, and to date, associations between increases in vaccination coverage and indirect impact from vaccination on gastroenteritis remain unclear. We aimed to assess the effect of rotavirus vaccination coverage on the number of all-age hospital inpatients with gastroenteritis in Japan. We also aimed to describe trends in the numbers of inpatients at high risk of severe gastroenteritis as a foundation for better understanding the public health impacts of infant rotavirus vaccination.

This study was approved by the Ethics Committee, Graduate School of Medicine, Kyoto University (approval no. R0135). Research was conducted in accordance with the Ethical Guidelines for Medical and Health Research Involving Human Subjects of the Ministry of Health, Labour, and Welfare, Japan.

## Methods

### Data Source

We used the Diagnosis Procedure Combination (DPC) administrative claims database, which is a case-mix classification system used in Japan for reimbursing acute care hospitals under the public medical insurance scheme (*25*). DPC administrative claims data include information on hospital codes, patient demographics, admission and discharge dates, admission routes, outcomes, primary and secondary diagnoses (based on codes from the International Classification of Diseases 10th Revision [ICD-10]), comorbidities, complications, and claims for medical services (*25*). We extracted DPC administrative claims data from the database of the DPC Study Group, comprising voluntarily participating hospitals, which account for >50% of all acute inpatients in Japan (*26*). We obtained data on rotavirus vaccination coverage by prefecture and year as actual measured values from a previous review of rotavirus vaccination in Japan (*17*). 

### Study Population and Definitions

We used data from hospitals that had continuously provided DPC data to the DPC Study Group during 2011–2019 to select the study population. We included in the study all hospitalization data that stated a primary diagnosis, diagnosis causing admission, or the most medically resource-intensive diagnosis of all-cause gastroenteritis during January 2011–December 2019. We used date of discharge, rather than date of admission, to determine the number of monthly inpatients because DPC data were generated after discharge. Gastroenteritis was defined by ICD-10 codes A08.x–A09.x. We categorized included patients into 4 age groups: <5, 5–19, 20–59, and ≥60 years. We defined patients as age-ineligible for rotavirus vaccination if they were age ≥1 year in 2011, ≥2 years in 2012, ≥3 years in 2013, ≥4 years in 2014, ≥5 years in 2015, ≥6 years in 2016, ≥7 years in 2017, ≥8 years in 2018, and ≥9 years in 2019. We obtained information on underlying conditions and medications of patients from the database. We defined underlying conditions at admission by ICD-10 codes C00.0-C97 for cancer and B20.x-B24 for HIV and defined immunocompromised patients as those with cancer or HIV or who had received steroids or immunosuppressants. We defined the rotavirus epidemic season as February–April, on the basis of data from previous studies (*27*,*28*).

### Statistical Analysis

Our primary outcome of interest was the monthly number of inpatients with all-cause gastroenteritis. We presented the results of time-series of monthly gastroenteritis hospitalizations in total, by age group, and among immunocompromised patients. We also presented annual gastroenteritis hospitalizations during the rotavirus epidemic season in total, by age group, and among immunocompromised patients. We estimated the impact of RV vaccination coverage on the basis of monthly number of gastroenteritis hospitalizations generalized estimating equations (GEE) (*29*–*31*) using a log-link function and Poisson distribution. We performed primary analyses using the GEE model for overall patients, the age-ineligible population, and immunocompromised patients. We further performed prespecified secondary analyses on data only from rotavirus-epidemic seasons. In our analyses, we did not account for repeated admission of the same patient, because we assumed that disease duration is short and recurrence rare among patients with gastroenteritis.

Because the numbers of gastroenteritis hospitalizations had a serial correlation within each hospital because of local characteristics and were therefore not independent, we designated hospital codes as cluster variables in the GEE models. We used an exchangeable correlation structure in the GEE model. We considered monthly gastroenteritis inpatients by hospital as a dependent variable and rotavirus vaccination coverage by prefecture as an independent variable. We included age groups, years, and months as covariates in the adjusted model. We treated rotavirus vaccination coverage by prefecture and year as categorical variables (based on incremental 10% increases in coverage), because we assumed that the relationship between coverage and the primary outcome was nonlinear. We considered coverage <40% to be the reference category because annual mean coverage reached as high as 30% in 2012, the second year of the study period. We also treated the 4 age groups and 12 month–defined groups as categorical variables. We calculated incidence rate ratios (IRRs) and 95% CIs from the GEE model. We considered p values <0.05 statistically significant; all tests were 2-tailed. We conducted statistical analyses using Stata/SE version 16.1 (StataCorp LLC, https://www.stata.com). 

## Results

We analyzed 294,108 hospitalizations for gastroenteritis from 569 hospitals in all 47 prefectures in Japan during 2011–2019. Among patient characteristics, median age was 41 years (interquartile range [IQR] 9–73 years), and the highest number (38.3%) of patients was in the ≥60-year age group. The proportion of immunocompromised patients was 12.94% (Table 1). 

**Table 1:** Characteristics of gastroenteritis patients in study of effects of rotavirus vaccination coverage among infants on hospital admission for gastroenteritis across all age groups, Japan, 2011–2019*

Characteristic	Value
Overall no. patients	294,108
Median age, y (IQR)	41 (9–73)
Age group, y	
<5	51,501 (17.5)
5–19	47,570 (16.2)
20–59	82,392 (28.0)
≥60	112,645 (38.3)
Immunocompromised patients	38,058 (12.9)
Underlying conditions	
Cancer	23,948 (8.1)
HIV infection	120 (0.04)
Medication	
Steroids	17,276 (5.9)
Immunosuppressants	3,459 (1.2)
Median stay, d (IQR)	5 (3–9)

*Values are no. (%) patients except as indicated. IQR, interquartile range

### Time-Series of Gastroenteritis Hospitalizations 

**Figure 1:**
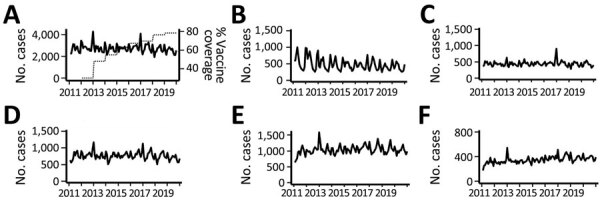
Time trends in gastroenteritis hospitalizations among different study populations in study of effects of rotavirus vaccination coverage among infants on hospital admission for gastroenteritis across all age groups, Japan, 2011–2019. A) Monthly numbers of gastroenteritis inpatients in the overall population, compared with annual mean rotavirus vaccination coverage. B–F) Monthly numbers of gastroenteritis hospitalizations among different study populations: B) young children <5 years of age; C) older children and adolescents 5–19 years of age; D) adults 20–59 years of age; E) older adults ≥60 years of age; F) immunocompromised persons.

**Figure 2:**
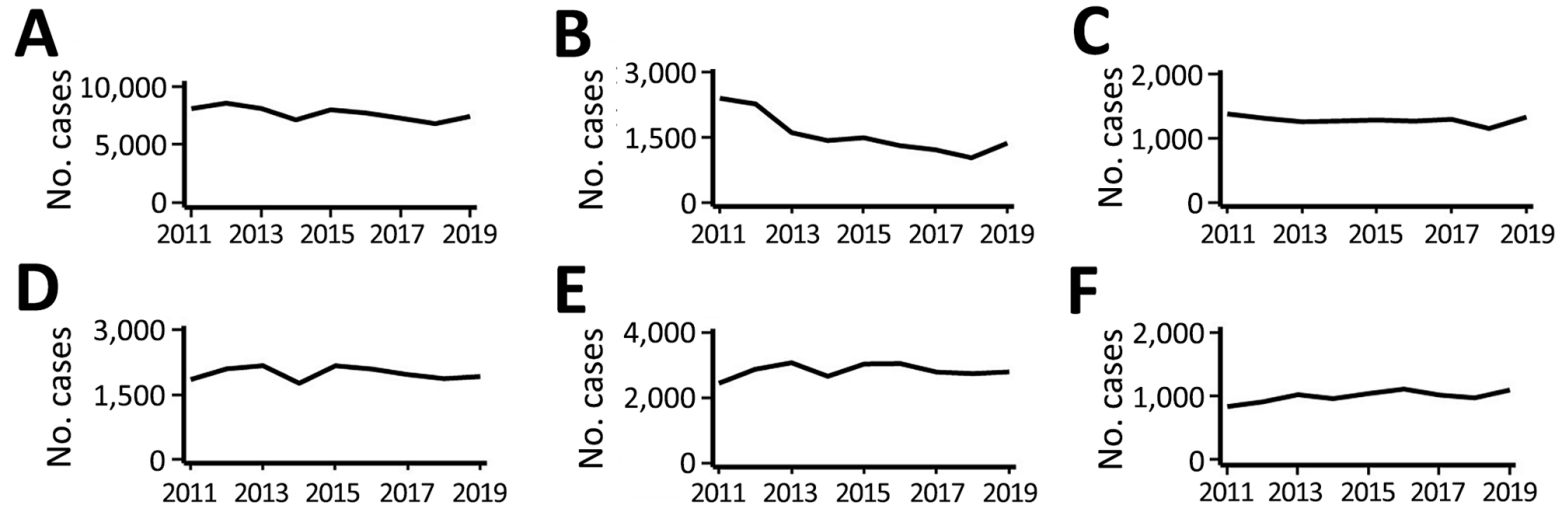
Annual numbers of gastroenteritis hospitalizations during the rotavirus vaccination epidemic season among different study populations in study of effects of rotavirus vaccination coverage among infants on hospital admission for gastroenteritis across all age groups, Japan, 2011–2019. A) Overall population; B) young children <5 years of age; C) older children and adolescents 5–19 years of age; D) adults 20–59 years of age; E) older adults ≥60 years of age; F) immunocompromised persons.

Annual mean rotavirus vaccination coverage in Japan increased from 30% in 2012 to 78% in 2019 (Figure 1, panel A) (*17*). Monthly numbers of gastroenteritis inpatients showed seasonality, peaking in winter (November–January). We observed epidemic peaks in December 2012 and December 2016. We found no obvious secular trends in overall gastroenteritis hospitalizations (Figure 1, panel A). We observed a trend of decreasing gastroenteritis hospitalizations among children <5 years of age in the first half of the study period (Figure 1, panel B), but numbers remained relatively stable among older children and adolescents (Figure 1, panel C) and young adults (Figure 1, panel D). We observed a secular trend of increased hospitalizations among adults ≥60 years of age (Figure 1, panel E) and among immunocompromised patients (Figure 1, panel F). Secular trends in the annual numbers of gastroenteritis hospitalizations during rotavirus epidemic seasons varied (Figure 2). We observed a decreasing trend in hospitalizations during rotavirus -epidemic seasons in young children (Figure 2, panel B) and an increasing trend in older adults (Figure 2, panel E) and immunocompromised patients (Figure 2, panel F). 

### Association of Vaccination Coverage with Monthly Numbers of Gastroenteritis Inpatients

**Figure 3:**
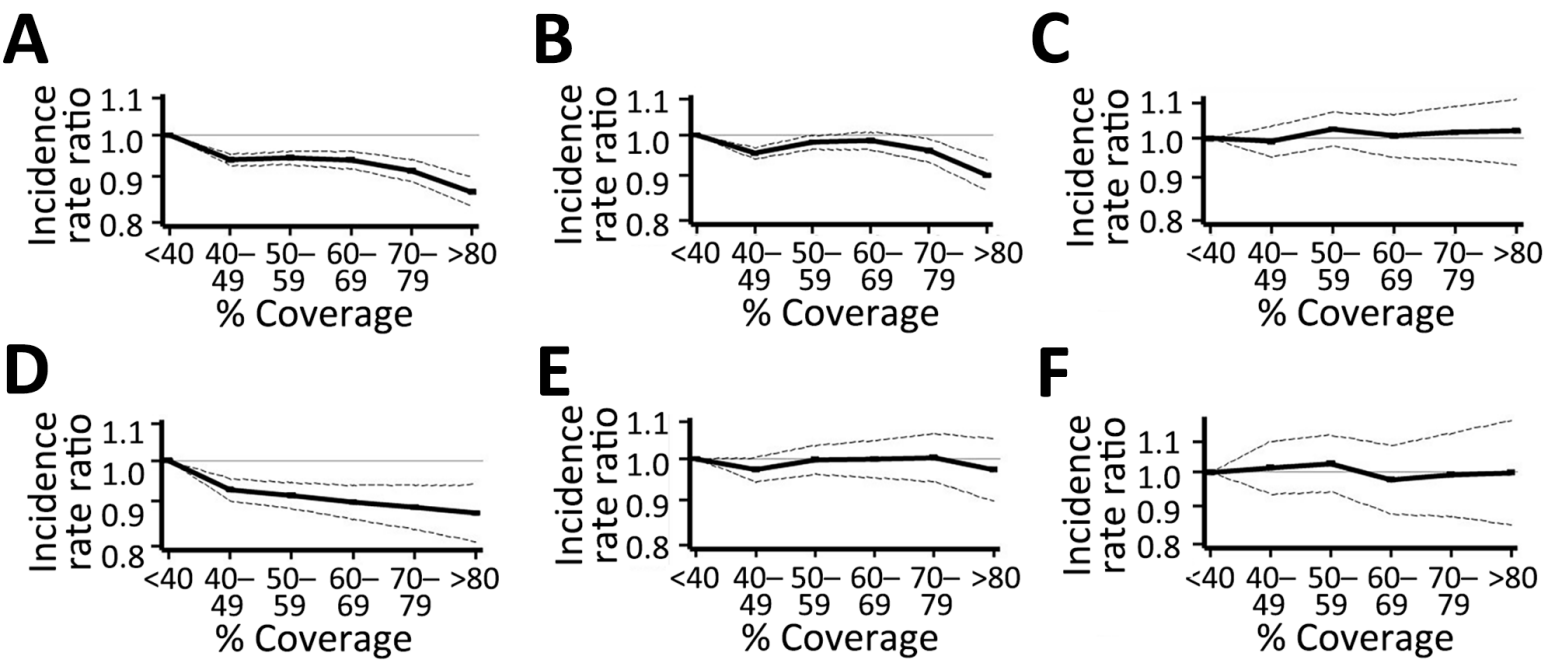
Association between vaccination coverage percentages and gastroenteritis hospitalization from generalized estimating equations analyses in study of effects of rotavirus vaccination coverage among infants on hospital admission for gastroenteritis across all age groups, Japan, 2011–2019. A–C) Annual associations for the overall population (A), the vaccination age-ineligible population (B), and immunocompromised persons (C). D–F) Associations during rotavirus epidemic seasons, February–April, for the overall population (D), vaccination age-ineligible population (E), and immunocompromised persons (F). Dashed lines indicate 95% CIs; gray horizontal lines indicate an incidence rate ratio of 1.0.

Among the overall patient population (N = 294,108), higher rotavirus vaccination coverage was associated with reduced monthly gastroenteritis hospitalizations compared with the reference category of <40% coverage. The IRR for gastroenteritis hospitalization was 0.87 (95% CI 0.83–0.90) for vaccination coverage of >80% after adjusting for age group, year, and month (Table 2; Figure 3, panel A). In the age-ineligible population for rotavirus vaccination (n = 247,156), the IRR for gastroenteritis hospitalization was also lower for vaccination coverage of >80% (adjusted IRR 0.90, 95% CI, 0.87–0.94), and the association between coverage and decreased monthly gastroenteritis hospitalizations was not consistently observed when coverage was <80%. (Figure 3, panel B). However, rotavirus vaccination coverage was not associated with changes in monthly gastroenteritis hospitalizations among immunocompromised patients (Figure 3, panel C). Secondary analyses restricted to rotavirus epidemic seasons also revealed associations between vaccination coverage and monthly gastroenteritis hospitalizations (Table 3). IRRs for gastroenteritis hospitalization gradually decreased with increasing vaccination coverage in the overall population (Figure 3, panel D). We found no vaccination coverage–related reduction in IRRs among the population age-ineligible for vaccination (Figure 3, panel E) or immunocompromised patients (Figure 3, panel F).

**Table 2:** Generalized estimating equations analyses to estimate the impact of rotavirus vaccination coverage on monthly number of gastroenteritis hospitalizations across all age groups, Japan, 2011–2019*

Vaccination coverage, %	IRR (95% CI)							
	Overall, N = 294,108			Age-ineligible for vaccination, n = 247,156			Immunocompromised, n = 38,058	
	Crude	Adjusted†		Crude	Adjusted†		Crude	Adjusted†
<40	Referent			Referent			Referent	
40–49	0.96 (0.95–0.97)	0.94 (0.93–0.95)		0.97 (0.96–0.98)	0.95 (0.94–0.97)		1.01 (0.97–1.04)	0.99 (0.95–1.03)
50–59	0.97 (0.96–0.98)	0.94 (0.93–0.96)		1.01 (0.99–1.02)	0.98 (0.96–1.00)		1.04 (1.01–1.08)	1.03 (0.98–1.07)
60–69	0.98 (0.97–0.99)	0.94 (0.92–0.96)		1.02 (1.00–1.03)	0.99 (0.96–1.01)		1.03 (1.00–1.07)	1.01 (0.95–1.07)
70–79	0.97 (0.96–0.98)	0.91 (0.89–0.94)		1.00 (0.99–1.01)	0.96 (0.93–0.99)		1.05 (1.02–1.09)	1.02 (0.95–1.09)
≥80	0.94 (0.92–0.95)	0.87 (0.83–0.90)		0.95 (0.93–0.96)	0.90 (0.87–0.94)		1.06 (1.02–1.11)	1.02 (0.93–1.12)

*IRR, incidence rate ratio. †Adjusted for age group, year, and month.

**Table 3:** Generalized estimating equations analyses, restricted to data from rotavirus epidemic seasons (February–April), to estimate the impact of rotavirus vaccination coverage on the monthly number of gastroenteritis hospitalizations across all age groups, Japan, 2011–2019*

Vaccination coverage, %	IRR (95% CI)							
	Overall, n = 69,025			Age-ineligible for vaccination, n = 56,605			Immunocompromised, n = 8,878	
	Crude	Adjusted†		Crude	Adjusted†		Crude	Adjusted†
<40	Referent			Referent			Referent	
40–49	0.93 (0.91–0.95)	0.93 (0.90–0.95)		0.96 (0.93–0.98)	0.97 (0.94–1.00)		1.03 (0.95–1.11)	1.01 (0.94–1.10)
50–59	0.92 (0.90–0.94)	0.91 (0.88–0.95)		0.98 (0.95–1.00)	1.00 (0.96–1.04)		1.05 (0.98–1.12)	1.03 (0.94–1.12)
60–69	0.91 (0.89–0.93)	0.90 (0.86–0.94)		0.96 (0.93–0.98)	1.00 (0.95–1.05)		1.01 (0.95–1.07)	0.98 (0.88–1.09)
70–79	0.90 (0.88–0.92)	0.89 (0.84–0.94)		0.94 (0.92–0.97)	1.00 (0.94–1.07)		1.03 (0.97–1.10)	0.99 (0.87–1.13)
≥80	0.89 (0.86–0.92)	0.87 (0.81–0.94)		0.90 (0.86–0.93)	0.97 (0.90–1.05)		1.04 (0.95–1.14)	1.00 (0.85–1.17)

*IRR, incidence rate ratio. †Adjusted for age group, year, and month.

## Discussion

Our study demonstrated the effect of rotavirus vaccination coverage on all-age gastroenteritis hospitalizations in Japan. Overall, we found that higher rotavirus vaccination coverage among infants was associated with lower monthly gastroenteritis hospitalizations for all age groups. We found a decreasing trend in IRRs for gastroenteritis hospitalizations as vaccination coverage increased in the overall population. Those results highlight the impact of coverage levels in understanding the effect of rotavirus vaccination on the entire population. The magnitude of this effect seems to be relatively small, however, with a point estimate of ≈0.9 for IRRs. We also showed trends in gastroenteritis hospitalizations among immunocompromised patients, who are at risk of severe gastroenteritis, after rotavirus vaccination introduction. Of note, rotavirus vaccination coverage was not associated with changes in monthly gastroenteritis hospitalizations among immunocompromised patients. Our results suggest that higher rotavirus vaccination coverage among infants has little effect on reducing gastroenteritis hospitalization in immunocompromised patients.

The findings of this study provide meaningful insights into the indirect protective benefits of rotavirus vaccination. Previous studies have suggested that infant rotavirus vaccination affords indirect protection against rotavirus in unvaccinated persons (*21*–*24*). Moreover, a multihospital discharge database study showed a lower gastroenteritis hospitalization rate among members of households in which a child had received the rotavirus vaccine (*32*). Infant rotavirus vaccination may contribute to reducing rotavirus transmission among unvaccinated persons by decreasing the probability of vaccine recipients becoming infected and likelihood of secondary transmission (*21*,*22*,*32*). In addition, the cumulative increase in rotavirus vaccine recipients might potentially lead to a reduction in rotavirus transmission in the entire population. The burden of rotavirus infection in adults compared with children is not well understood (*33*). Recent reports have highlighted the substantial magnitude of rotavirus in adults with gastroenteritis (*6*). Furthermore, several large studies have shown reductions in adult hospitalization after vaccination introduction (*23*,*24*), although the most affected age group varied among different cohorts, and findings in relation to indirect protection are inconsistent for adults. Taken together, those findings might provide supporting evidence for routine rotavirus immunization of infants and additional clues for understanding disease transmission.

Among major strengths of this study were use of 9-year longitudinal data from >500 hospitals and use of rotavirus vaccination coverage data at the regional level. Several studies of pneumococcal conjugate vaccine have investigated the association between vaccination coverage and indirect protection in the unvaccinated population (*34*,*35*). A recent study in Spain found an association between reductions in rotavirus gastroenteritis hospitalizations and regional rotavirus vaccination coverage in children <5 years of age (*36*). Those studies suggested that use of vaccination coverage data can provide valuable insights into understanding regional variations in vaccination coverage and associations between vaccination coverage and indirect protection. An additional strength of this study was its description of the postvaccination trends in hospitalizations for gastroenteritis among immunocompromised patients. 

Among our study’s limitations was an inherent problem with the diagnostic coding of gastroenteritis. Previous studies have shown the relatively low sensitivity in diagnosing gastroenteritis using ICD codes, both in children and adults (*37*,*38*). Possible misclassification of the diagnosis might have resulted in underestimation of the incidence rate of gastroenteritis. The effect of this limitation appears to be mitigated, however, because it is likely that any misclassification occurred equally across different levels of vaccination coverage. Another limitation was that we did not investigate the effect of vaccination coverage on rotavirus gastroenteritis hospitalization. We presumed that an analysis restricted only to patients with the diagnosis of rotavirus gastroenteritis would result in further underestimation of incidence because patients with gastroenteritis are typically not routinely tested for rotavirus in clinical settings (*39*,*40*). Instead, we performed secondary analyses with a restriction to the rotavirus epidemic season, which demonstrated results consistent with those of the primary analysis.

Another notable limitation of this study was that rotavirus vaccination coverage was obtained at an aggregate level. Heterogeneity in the distribution of vaccination coverage within the prefecture may lead to confounding for the observed association. Thus, to avoid the ecologic fallacy (*41*), our results should be interpreted with caution. In addition, we lacked information on socioeconomic status, which could affect both childhood vaccination coverage (*42*,*43*) and hospitalization rates. Although we used hospital codes as a cluster variable in the GEE models, results do not account for unmeasured confounding factors. The study population selected from hospitals in the DPC Study Group might have introduced selection bias, another potential limitation. The effect of this bias seems to be limited, however, given the broad coverage of the DPC Study Group database in acute inpatient care in Japan (*25*). 

In conclusion, our study assessed the effects of infant rotavirus vaccination coverage on the number of all-age gastroenteritis hospitalizations in Japan and found higher rotavirus vaccination coverage was associated with a decline in gastroenteritis hospitalization in the overall population. However, the effect size was relatively small. Our study also found gastroenteritis hospitalizations among immunocompromised patients were not affected after rotavirus vaccination introduction. Our findings shed light on the importance of rotavirus vaccination coverage and suggest that, by reducing gastroenteritis hospitalizations, rotavirus vaccination among infants might benefit the entire population. 

## References

[1] Bányai K, Estes MK, Martella V, Parashar UD. Viral gastroenteritis. Lancet. 2018;392:175–86. 10.1016/S0140-6736(18)31128-0 30025810 PMC8883799

[2] Hartman S, Brown E, Loomis E, Russell HA. Gastroenteritis in Children. Am Fam Physician. 2019;99:159–65. 30702253

[3] Bresee JS, Marcus R, Venezia RA, Keene WE, Morse D, Thanassi M, et al.; US Acute Gastroenteritis Etiology Study Team. The etiology of severe acute gastroenteritis among adults visiting emergency departments in the United States. J Infect Dis. 2012;205:1374–81. 10.1093/infdis/jis206 22454468

[4] Cardemil CV, Balachandran N, Kambhampati A, Grytdal S, Dahl RM, Rodriguez-Barradas MC, et al. Incidence, etiology, and severity of acute gastroenteritis among prospectively enrolled patients in 4 Veterans Affairs hospitals and outpatient centers, 2016–2018. Clin Infect Dis. 2021;73:e2729–38. 10.1093/cid/ciaa806 32584956 PMC9195496

[5] Lopman BA, Hall AJ, Curns AT, Parashar UD. Increasing rates of gastroenteritis hospital discharges in US adults and the contribution of norovirus, 1996-2007. Clin Infect Dis. 2011;52:466–74. 10.1093/cid/ciq163 21258098

[6] Troeger C, Blacker BF, Khalil IA, Rao PC, Cao S, Zimsen SRM, et al.; GBD 2016 Diarrhoeal Disease Collaborators. Estimates of the global, regional, and national morbidity, mortality, and aetiologies of diarrhoea in 195 countries: a systematic analysis for the Global Burden of Disease Study 2016. Lancet Infect Dis. 2018;18:1211–28. 10.1016/S1473-3099(18)30362-1 30243583 PMC6202444

[7] Vos T, Lim SS, Abbafati C, Abbas KM, Abbasi M, Abbasifard M, et al.; GBD 2019 Diseases and Injuries Collaborators. Global burden of 369 diseases and injuries in 204 countries and territories, 1990-2019: a systematic analysis for the Global Burden of Disease Study 2019. Lancet. 2020;396:1204–22. 10.1016/S0140-6736(20)30925-9 33069326 PMC7567026

[8] Tate JE, Burton AH, Boschi-Pinto C, Steele AD, Duque J, Parashar UD; WHO-coordinated Global Rotavirus Surveillance Network. 2008 estimate of worldwide rotavirus-associated mortality in children younger than 5 years before the introduction of universal rotavirus vaccination programmes: a systematic review and meta-analysis. Lancet Infect Dis. 2012;12:136–41. 10.1016/S1473-3099(11)70253-5 22030330

[9] Chen Y, Liu BC, Glass K, Kirk MD. High incidence of hospitalisation due to infectious gastroenteritis in older people associated with poor self-rated health. BMJ Open. 2015;5:e010161. 10.1136/bmjopen-2015-010161 26719326 PMC4710819

[10] Schmidt-Hieber M, Bierwirth J, Buchheidt D, Cornely OA, Hentrich M, Maschmeyer G, et al.; AGIHO Working Group. Diagnosis and management of gastrointestinal complications in adult cancer patients: 2017 updated evidence-based guidelines of the Infectious Diseases Working Party (AGIHO) of the German Society of Hematology and Medical Oncology (DGHO). Ann Hematol. 2018;97:31–49. 10.1007/s00277-017-3183-7 29177551 PMC5748412

[11] Balachandran N, Cates J, Kambhampati AK, Marconi VC, Whitmire A, Morales E, et al. Risk factors for acute gastroenteritis among patients hospitalized in 5 Veterans Affairs medical centers, 2016–2019. Open Forum Infect Dis. 2022;9:ofac339. 10.1093/ofid/ofac339 35949407 PMC9356693

[12] van Dongen JAP, Rouers EDM, Schuurman R, Bonten MJM, Bruijning-Verhagen P; RIVAR Study Group. Acute gastroenteritis disease burden in infants with medical risk conditions in the Netherlands. Pediatr Infect Dis J. 2021;40:300–5. 10.1097/INF.0000000000003002 33230056 PMC7952044

[13] Moon RC, Bleak TC, Rosenthal NA, Couturier B, Hemmert R, Timbrook TT, et al. Epidemiology and economic burden of acute infectious gastroenteritis among adults treated in outpatient settings in US health systems. Am J Gastroenterol. 2023;118:1069–79. 10.14309/ajg.0000000000002186 36728224 PMC10226474

[14] Arakaki L, Tollefson D, Kharono B, Drain PK. Prevalence of rotavirus among older children and adults with diarrhea: A systematic review and meta-analysis. Vaccine. 2021;39:4577–90. 10.1016/j.vaccine.2021.06.073 34244008

[15] Tate JE, Burton AH, Boschi-Pinto C, Parashar UD; World Health Organization–Coordinated Global Rotavirus Surveillance Network. Global, regional, and national estimates of rotavirus mortality in children <5 years of age, 2000–2013. Clin Infect Dis. 2016;62(Suppl 2):S96–105. 10.1093/cid/civ1013 27059362 PMC11979873

[16] World Health Organization. Rotavirus vaccines:an update. Wkly Epidemiol Rec. 2009;84:533–40. 20034143

[17] Tsugawa T, Akane Y, Honjo S, Kondo K, Kawasaki Y. Rotavirus vaccination in Japan: Efficacy and safety of vaccines, changes in genotype, and surveillance efforts. J Infect Chemother. 2021;27:940–8. 10.1016/j.jiac.2021.04.002 33867267

[18] Kobayashi M, Adachi N, Miyazaki M, Tatsumi M. Decline of rotavirus-coded hospitalizations in children under 5 years: A report from Japan where rotavirus vaccines are self-financed. Vaccine. 2018;36:2727–32. 10.1016/j.vaccine.2017.10.033 29241644

[19] Aliabadi N, Antoni S, Mwenda JM, Weldegebriel G, Biey JNM, Cheikh D, et al. Global impact of rotavirus vaccine introduction on rotavirus hospitalisations among children under 5 years of age, 2008-16: findings from the Global Rotavirus Surveillance Network. Lancet Glob Health. 2019;7:e893–903. 10.1016/S2214-109X(19)30207-4 31200889 PMC7336990

[20] Mwenda JM, Hallowell BD, Parashar U, Shaba K, Biey JN, Weldegebriel GG, et al. Impact of rotavirus vaccine introduction on rotavirus hospitalizations among children under 5 years of age—World Health Organization African Region, 2008–2018. Clin Infect Dis. 2021;73:1605–8. 10.1093/cid/ciab520 34089588 PMC11703079

[21] Payne DC, Staat MA, Edwards KM, Szilagyi PG, Weinberg GA, Hall CB, et al.; New Vaccine Surveillance Network (NVSN). Direct and indirect effects of rotavirus vaccination upon childhood hospitalizations in 3 US Counties, 2006-2009. Clin Infect Dis. 2011;53:245–53. 10.1093/cid/cir307 21705316

[22] Anderson EJ, Shippee DB, Weinrobe MH, Davila MD, Katz BZ, Reddy S, et al. Indirect protection of adults from rotavirus by pediatric rotavirus vaccination. Clin Infect Dis. 2013;56:755–60. 10.1093/cid/cis1010 23349228

[23] Wilson SE, Rosella LC, Wang J, Renaud A, Le Saux N, Crowcroft NS, et al. Equity and impact: Ontario’s infant rotavirus immunization program five years following implementation. A population-based cohort study. Vaccine. 2019;37:2408–14. 10.1016/j.vaccine.2019.01.061 30765171

[24] Baker JM, Tate JE, Steiner CA, Haber MJ, Parashar UD, Lopman BA. Longer-term direct and indirect effects of infant rotavirus vaccination across all ages in the United States in 2000–2013: analysis of a large hospital discharge data set. Clin Infect Dis. 2019;68:976–83. 10.1093/cid/ciy580 30020438 PMC7182126

[25] Hayashida K, Murakami G, Matsuda S, Fushimi K. History and profile of diagnosis procedure combination (DPC): development of a real data collection system for acute inpatient care in Japan. J Epidemiol. 2021;31:1–11. 10.2188/jea.JE20200288 33012777 PMC7738645

[26] Yasunaga H. Real world data in Japan: Chapter II. The diagnosis procedure combination database. Ann Clin Epidemiolol. 2019;1:76–79.

[27] Suzuki H, Sakai T, Tanabe N, Okabe N. Peak rotavirus activity shifted from winter to early spring in Japan. Pediatr Infect Dis J. 2005;24:257–60. 10.1097/01.inf.0000154327.00232.4d 15750463

[28] Tajiri H, Takeuchi Y, Takano T, Ohura T, Inui A, Yamamoto K, et al. The burden of rotavirus gastroenteritis and hospital-acquired rotavirus gastroenteritis among children aged less than 6 years in Japan: a retrospective, multicenter epidemiological survey. BMC Pediatr. 2013;13:83. 10.1186/1471-2431-13-83 23697664 PMC3664221

[29] Liang KY, Zeger SL. Longitudinal data analysis using generalized linear models. Biometrika. 1986;73:13–22. 10.1093/biomet/73.1.13

[30] Yarnell CJ, Fu L, Manuel D, Tanuseputro P, Stukel T, Pinto R, et al. Association between immigrant status and end-of-life care in Ontario, Canada. JAMA. 2017;318:1479–88. 10.1001/jama.2017.14418 28973088 PMC5710367

[31] Insaf TZ, Sommerhalter KM, Jaff TA, Farr SL, Downing KF, Zaidi AN, et al. Access to cardiac surgery centers for cardiac and non-cardiac hospitalizations in adolescents and adults with congenital heart defects- a descriptive case series study. Am Heart J. 2021;236:22–36. 10.1016/j.ahj.2021.02.018 33636136 PMC8097661

[32] Cortese MM, Dahl RM, Curns AT, Parashar UD. Protection against gastroenteritis in US households with children who received rotavirus vaccine. J Infect Dis. 2015;211:558–62. 10.1093/infdis/jiu503 25234721

[33] Karakusevic A, Devaney P, Enstone A, Kanibir N, Hartwig S, Carias CDS. The burden of rotavirus-associated acute gastroenteritis in the elderly: assessment of the epidemiology in the context of universal childhood vaccination programs. Expert Rev Vaccines. 2022;21:929–40. 10.1080/14760584.2022.2066524 35535677

[34] Chan J, Gidding HF, Blyth CC, Fathima P, Jayasinghe S, McIntyre PB, et al. Levels of pneumococcal conjugate vaccine coverage and indirect protection against invasive pneumococcal disease and pneumonia hospitalisations in Australia: An observational study. PLoS Med. 2021;18:e1003733. 10.1371/journal.pmed.1003733 34343186 PMC8376256

[35] Oyewole OR, Lang P, Albrich WC, Wissel K, Leib SL, Casanova C, et al. The impact of pneumococcal conjugate vaccine (PCV) coverage heterogeneities on the changing epidemiology of invasive pneumococcal disease in Switzerland, 2005–2019. Microorganisms. 2021;9:1078. 10.3390/microorganisms9051078 34069761 PMC8157260

[36] Ruiz-Contreras J, Alfayate-Miguelez S, Carazo-Gallego B, Onís E, Díaz-Munilla L, Mendizabal M, et al. Rotavirus gastroenteritis hospitalizations in provinces with different vaccination coverage rates in Spain, 2013-2018. BMC Infect Dis. 2021;21:1138. 10.1186/s12879-021-06841-x 34742235 PMC8572461

[37] Hsu VP, Staat MA, Roberts N, Thieman C, Bernstein DI, Bresee J, et al. Use of active surveillance to validate international classification of diseases code estimates of rotavirus hospitalizations in children. Pediatrics. 2005;115:78–82. 10.1542/peds.2004-0860 15629984

[38] Pindyck T, Hall AJ, Tate JE, Cardemil CV, Kambhampati AK, Wikswo ME, et al. Validation of acute gastroenteritis–related International Classification of Diseases, Clinical Modification Codes in pediatric and adult US populations. Clin Infect Dis. 2020;70:2423–7. 10.1093/cid/ciz846 31626687 PMC7390357

[39] Patel MM, Tate JE, Selvarangan R, Daskalaki I, Jackson MA, Curns AT, et al. Routine laboratory testing data for surveillance of rotavirus hospitalizations to evaluate the impact of vaccination. Pediatr Infect Dis J. 2007;26:914–9. 10.1097/INF.0b013e31812e52fd 17901797

[40] Cardemil CV, O’Leary ST, Beaty BL, Ivey K, Lindley MC, Kempe A, et al. Primary care physician knowledge, attitudes, and diagnostic testing practices for norovirus and acute gastroenteritis. PLoS One. 2020;15:e0227890. 10.1371/journal.pone.0227890 31935271 PMC6959576

[41] Wakefield J. Ecologic studies revisited. Annu Rev Public Health. 2008;29:75–90. 10.1146/annurev.publhealth.29.020907.090821 17914933

[42] Pearce A, Law C, Elliman D, Cole TJ, Bedford H; Millennium Cohort Study Child Health Group. Factors associated with uptake of measles, mumps, and rubella vaccine (MMR) and use of single antigen vaccines in a contemporary UK cohort: prospective cohort study. BMJ. 2008;336:754–7. 10.1136/bmj.39489.590671.25 18309964 PMC2287222

[43] Kuroda H, Goto A, Kawakami C, Yamamoto K, Ito S, Kamijima M, et al.; Japan Environment and Children’s Study (JECS) Group. Association between a single mother family and childhood undervaccination, and mediating effect of household income: a nationwide, prospective birth cohort from the Japan Environment and Children’s Study (JECS). BMC Public Health. 2022;22:117. 10.1186/s12889-022-12511-7 35038996 PMC8764848

